# Hawk Eyes I: Diurnal Raptors Differ in Visual Fields and Degree of Eye Movement

**DOI:** 10.1371/journal.pone.0012802

**Published:** 2010-09-22

**Authors:** Colleen T. O'Rourke, Margaret I. Hall, Todd Pitlik, Esteban Fernández-Juricic

**Affiliations:** 1 Department of Biological Sciences, California State University Long Beach, Long Beach, California, United States of America; 2 Department of Physiology, Midwestern University, Glendale, Arizona, United States of America; 3 Department of Agriculture - Animal and Plant Health Inspection Service - Wildlife Services, Los Angeles, California, United States of America; 4 Department of Biological Sciences. Purdue University, West Lafayette, Indiana, United States of America; Lund University, Sweden

## Abstract

**Background:**

Different strategies to search and detect prey may place specific demands on sensory modalities. We studied visual field configuration, degree of eye movement, and orbit orientation in three diurnal raptors belonging to the Accipitridae and Falconidae families.

**Methodology/Principal Findings:**

We used an ophthalmoscopic reflex technique and an integrated 3D digitizer system. We found inter-specific variation in visual field configuration and degree of eye movement, but not in orbit orientation. Red-tailed Hawks have relatively small binocular areas (∼33°) and wide blind areas (∼82°), but intermediate degree of eye movement (∼5°), which underscores the importance of lateral vision rather than binocular vision to scan for distant prey in open areas. Cooper's Hawks' have relatively wide binocular fields (∼36°), small blind areas (∼60°), and high degree of eye movement (∼8°), which may increase visual coverage and enhance prey detection in closed habitats. Additionally, we found that Cooper's Hawks can visually inspect the items held in the tip of the bill, which may facilitate food handling. American Kestrels have intermediate-sized binocular and lateral areas that may be used in prey detection at different distances through stereopsis and motion parallax; whereas the low degree eye movement (∼1°) may help stabilize the image when hovering above prey before an attack.

**Conclusions:**

We conclude that: (a) there are between-species differences in visual field configuration in these diurnal raptors; (b) these differences are consistent with prey searching strategies and degree of visual obstruction in the environment (e.g., open and closed habitats); (c) variations in the degree of eye movement between species appear associated with foraging strategies; and (d) the size of the binocular and blind areas in hawks can vary substantially due to eye movements. Inter-specific variation in visual fields and eye movements can influence behavioral strategies to visually search for and track prey while perching.

## Introduction

Foraging specializations are the result of morphological and metabolic capabilities of foragers, food availability, habitat structure, etc. [1], [2]. Furthermore, in the case of visually oriented organisms like birds, sensory specializations may help gather visual information necessary to detect prey against the background and track them visually until capture. For instance, the retinas of some sea birds have long visual streaks, which are areas with high retinal ganglion cell density that provide high visual resolution along the horizon [3], to enhance food detection from the distance [4]. Upon detection, individuals visually track the prey target by flying over it and changing their head movement patterns so that the visual streak is aligned with the prey item [5].

Specific foraging strategies allow individuals to improve the chances of prey detection and capture under certain ecological conditions. These foraging strategies require the use of different behaviors (gleaning, sallying, hovering, etc. [6]) that are influenced by food availability and micro-habitat structure [7], [8]. Different types of foraging strategies are expected to place different demands on the visual systems. This can be particularly relevant for species that capture active prey as greater visual capacity is required for detecting and chasing moving prey targets [9]. For example, sit-and-wait predation requires detecting prey at a distance before engaging in an attack [10], as opposed to probing, whereby after the bill is inserted into the substrate and opened, eyes are swung forward to detect prey items at close distances [11]. Furthermore, the success of certain foraging techniques can increase with the degree of visual coverage. For instance, herons have a large vertical extent of their binocular fields below the bill to enhance the chances of detecting and then capturing highly evasive prey with a single strike [12].

We asked whether diurnal birds of prey (hereafter: “raptors”) with different foraging strategies vary in visual traits (visual fields, degree of eye movement, orbit convergence) relevant to gathering visual information used in prey searching. We studied Red-tailed Hawks *Buteo jamaicensis*, Cooper's Hawks *Accipiter cooperi*, and American Kestrels *Falco sparverius*.

Red-tailed Hawks are large (1,126 g [13]) sit-and-wait predators that hunt ground-dwelling mammals, reptiles and birds, generally by perching on high, exposed perches and scanning open habitats [14]–[16]. Cooper's Hawks are medium-sized active-ambushing predators (439 g [13]) that live in forested habitats, and most frequently hunt birds and tree-dwelling mammals by chasing prey through forest and brush [14], [15], [17]. American Kestrels are small (115 g [13]) falcons that preferentially hunt in open habitats small mammals and large insects from perches or by hovering and then stooping down onto prey [18]. Because of their small size, American Kestrels are also sometimes subject to predation by larger diurnal raptors, owls, and corvids [14], [15], [18]. Red-tailed Hawks and Cooper's Hawks are both in the Family Accipitridae, and American Kestrels in the Family Falconidae [19].

The visual field defines the amount of space around the head from which an individual can potentially gather visual information at any one instant [20]. Visual fields vary, as a result of foraging and predation pressures [21], in the relative size of the (a) binocular area (involved in prey handling, feeding chicks, etc.), (b) lateral area (which generally encompasses the fovea, the retinal area with the highest acuity, [22]), and (c) blind area (which has no visual coverage). The relative sizes of these three visual field components can be good indicators of sensory adaptations to environmental conditions. The visual fields of predators have received comparatively less attention [20], [23] than those of their prey species [24], [25], [26]. For instance, the binocular fields of a diurnal raptor (Short-toed Eagle [20]) are small in width (20°) and vertical extent (80°), but they are wider (48°) in a nocturnal raptor (Tawny Owl [23]). The orientation of the orbit in the skull can influence the size of the binocular field, such that species with orbits that tend to converge towards the frontal part of the skull are expected to have wider binocular fields [26], [27]. Additionally, the role of eye movements can be important not only to track objects visually, but also to modify visual coverage. Some species with relatively wide blind areas above and behind their heads could compensate for the reduced visual coverage by using wide amplitude eye movements to diverge the eyes towards the rear of the heads [26]. However, the degree of this type of eye movement varies considerably between species [11], [26], [28], [29], and in general raptors are not characterized by a large degree of eye movements (e.g., Short-toed Eagle *Circaetus gallicus* [20]; Tawny Owl *Strix aluco* [23]).

Understanding raptor vision and behavior can shed light into the diversity of predator hunting strategies and potentially the evolution of anti-predator behavior in birds [30], [31], as prey may benefit from avoidance behaviors that exploit a constraint in the visual system of the predator. For example, in owls, narrow lateral visual fields and low degree of eye movement may reduce their hunting success when prey move sideways from the line of attack [32]. Our study focused on the predator's point of view by comparing the visual fields, degree of eye movement, and orbit convergence between three diurnal raptors.

## Methods

### Visual fields and eye movements

**Figure 1:**
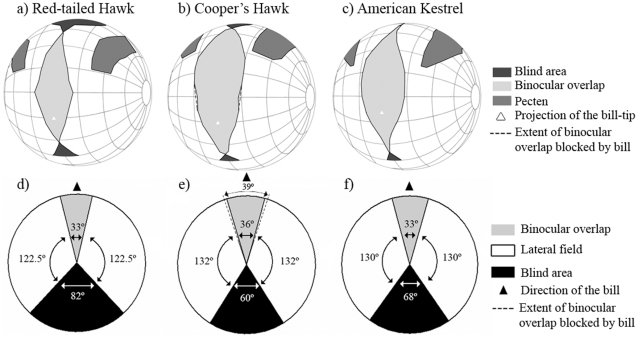
Visual fields of three diurnal raptors with the eyes at rest. Two views of the visual fields of Red-tailed Hawks (a, d), Cooper's Hawks (b, e), and American Kestrels (c, f). (a–c) Orthographic projection of the boundaries of the retinal fields of the two eyes, along with projection of the pectens and bill tips. A latitude and longitude coordinate system was used with the equator aligned vertically in the median sagittal plane. The bird's head is imagined to be at the center of the globe (grid is at 20° intervals). (d–f) Horizontal sections through the horizontal plane (90°–270°) showing the visual field configuration of each species. Each chart represents the average retinal visual field when the eyes were at rest. The dotted lines in the Cooper's Hawk representations (b, e) represent extrapolated binocular field assuming that the retinal margin follows a circular projection (see text).

**Figure 2:**
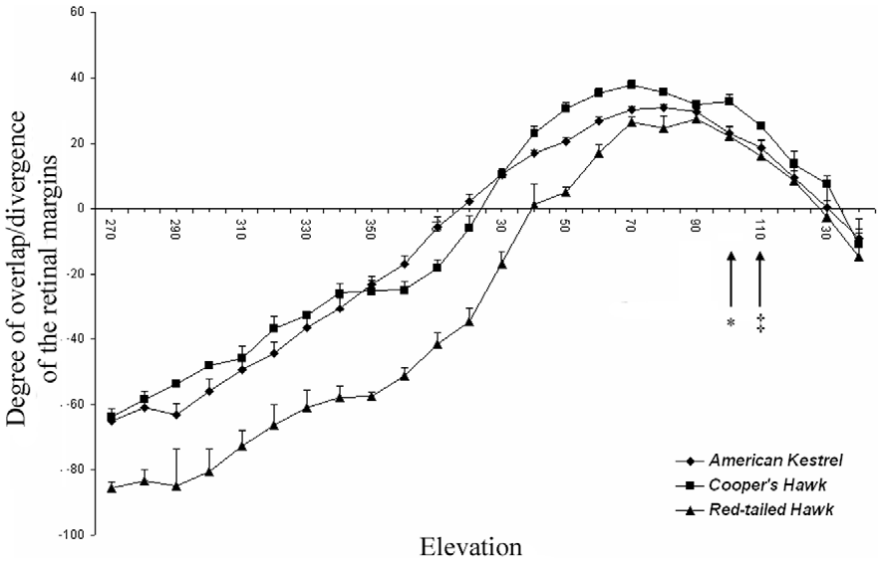
Binocular overlap and blind areas across elevations around the head of three diurnal raptors. Mean (± SE) angular separation of the retinal field margins as a function of elevation in the median sagittal plane in Red-tailed Hawks, Cooper's Hawks, and American Kestrels. Binocular fields are indicated by positive values of overlap of the visual field margins; whereas blind areas are indicated by negative values. The horizontal plane is represented by 90° (front of the head) to 270° (back of the head), with 0° indicating a position above the head. Arrows indicate projection of the bill-tip (‡  =  Cooper's Hawk and Red-tailed Hawk; *  =  American Kestrel).

**Figure 3:**
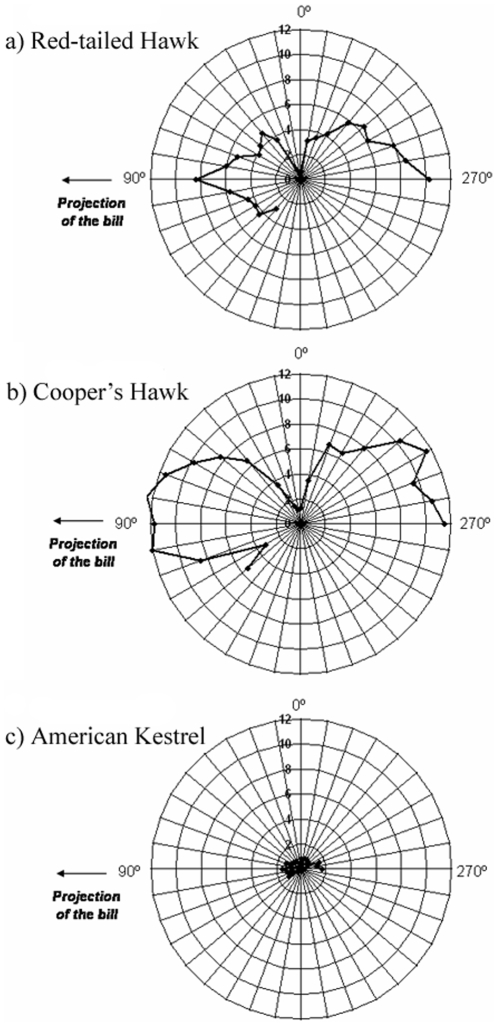
Eye movements in three diurnal raptors. Average degree of eye movements as a function of elevation in the median sagittal plane in (a) Red-tailed Hawks, (b) Cooper's Hawks, and (c) American Kestrels. Degree of eye movement is shown in the same scale (0 – 12°) in all species. Elevations are envisioned as if viewing the head of the bird from the left side, with the bill projecting at approximately 90°.

The raptors used in this study were obtained with the cooperation of the U.S. Department of Agriculture, Animal and Plant Health Inspection Service (USDA-APHIS) Wildlife Services, Agency personnel caught the raptors from a number of locations in Los Angeles County, CA (Federal Fish and Wildlife Permit #MB004760-0). After capture, individuals were brought to the lab for less than 2 hrs to measure their visual fields with the collaboration of USDA personnel, and later transported to a state-approved raptor rehabilitation facility for relocation. All animal handling procedures for this project were approved by California State University Long Beach Institution Animal Care and Use Committee (protocol #256),

Measurements were taken using a visual field apparatus [23]. Individuals were restrained in the center of the apparatus in a horizontal position. Bills were positioned in wire-based beak holders. We used an angular coordinate system to measure the configuration of the visual field. The head of the bird lies at the center of a space defined as a globe, with the horizontal axis of the globe traveling through both eyes. The 0° elevation lies directly above the head of the bird, and elevations increase in 10° increments around the bird, such that 90° lies directly in front of the bird's head, and 270° lies directly behind the bird's head at the horizontal plane (see example of coordinate system in Figs. 1, 2, 3). The head was held at the angle birds naturally assume, based on pictures of perching individuals. Previous visual field studies have generally calculated only one angle to define the plane of the bill within the coordinate system [21]. However, because of the unusual shape of raptor bills (with the curved maxilla extending far below the lower mandible) we defined two separate measurements to model the bill in the coordinate system. The plane of the bill (a horizontal plane bisecting the eye and the tip of the lower mandible when the bird is in a resting position) for all three species was at the 90° elevation. The angle of the bill-tip (the angle from the eye to the tip of the maxilla) for each species was as follows: Red-tailed Hawks, 110°; Cooper's Hawks, 110°; American Kestrels, 100°.

We measured the retinal visual field with an opthalmoscopic reflex technique [23]. Using a Keeler Professional ophthalmoscope, we measured the retinal margins of each eye at each elevation in 10° increments, to an accuracy of ±0.5°, with measurements taken from 150° to 270° due to obstructions of the apparatus or the animal's body. Obstructions at some elevations are usual given the configuration of the visual field apparatus. Measurements were mathematically adjusted to correct for close viewing and represent a hypothetical infinite distant view point [23]. We measured visual fields with two different procedures: (1) when eyes were at rest, and (2) when eyes were converged and diverged. Sometimes the same individual was exposed to both methods. Sample sizes differed among species due to the unpredictable availability of individuals.

In the first method (eyes at rest), we measured the visual fields when the eyes of the individual were not visibly moving around or tracking the motion of the ophthalmoscope. We also measured the projection of the pecten, which is a vascular structure in the retina that projects a blind area in the visual field [22]. We measured six American Kestrels, seven Cooper's Hawks, and three Red-tailed Hawks. In the second method, we elicited eye movements with quick sounds and/or flashes of light directed at the front or the rear of the bird's head. With the eyes converged to or diverged from the bill, we recorded the maximum and minimum positions of the retinal field margins, the difference representing the degree of eye movement in a given plane. We also calculated the maximum and minimum size of the binocular, lateral, cyclopean (binocular + lateral right + lateral left visual fields), and blind areas. The lateral field (monocular field – binocular field) was calculated as: (360- (mean blind field + mean binocular field)/2), following Fernández-Juricic et al. [26]. We measured ten American Kestrels, four Cooper's Hawks, and three Red-tailed Hawks.

### Orbit orientation

Orbit orientation was measured on skeletal specimens (three specimens per species) obtained from the California State University Long Beach Vertebrate Museum (Long Beach, CA) and the American Museum of Natural History (New York, NY). All specimens came from populations in Southern California, but one Cooper's Hawk came from New York State. Each specimen was measured three times and the means calculated to obtain a species value. Measures of the orbital and median sagittal planes of the skull were calculated using three-dimensional coordinates. Six landmark points on the skull (three for each plane) were measured with a Micro-Scribe-3DX coordinate data stylus (Immersion Corp., Can Jose, CA, USA). The median sagittal plane is a vertical plane bisecting the skull lengthwise, defined by three points: (1) the anterior-most point of the beak, (2) the point where the internasal suture meets the inter-premaxillary suture, and (3) the posterior-most projection of the skull. Three points defined the orbital plane: (1) the mid-point on the quadratojugular bar (*orbitale inferius*), (2) the point on the orbital margin directly opposite and furthest from point (1) (*orbitale superius*), and (3) the central point of the lacrimal bone and the point furthest from the posterior-most projection of the skull (*orbitale anterius*) [27]. Orbit convergence was defined as the dihedral angle between the orbital plane and the median sagittal plane. For a full description of how orbit convergence was calculated, see [27]. Briefly, orbit convergence was calculated from the coordinate data following a standard trigonometric function for dihedral angle computation (e.g. [33]). A macro for this calculation is available in [34]. Higher values of orbit convergence indicate that the plane of the orbit deviates further from the sagittal plane, which means that the orbits face more towards the front of the skull.

### Statistical analysis

We used general linear models to assess differences among species in the following response factors: (a) overall width of the binocular field at rest (taking into account all elevations around the head, without eye movement), (b) overall width of the blind area at rest, (c) overall width of pecten, (d) overall vertical extent of the binocular field in the median sagittal plane, and (e) degree of eye movements. Besides a species factor, we included in the model elevation and the interaction between elevation and species to establish whether differences among species would change at different elevations around the head (Fig. 3 shows an example of the coordinate system used). We considered the estimates of these response factors at different elevations around the head for a given individual as repeated measures.

T-tests were used for pair-wise comparisons, from which we report the significant ones. We present means (± SE) throughout.

## Results

### At rest visual fields

Three-dimensional representations of the at-rest visual fields show that all species have their bill-tips projecting into the binocular field and have blind areas (Fig.1a–c). However, in the Cooper's Hawk the bill intruded enough in the binocular area to limit our measurements (Fig. 1b), which suggests that individuals can observe their bill tips [35]. The size of the binocular field at rest at elevation 90° was estimated as 39° for the Cooper's Hawk (Fig. 1e). This estimate was extrapolated from elevations right above and below 90°, assuming that the retinal margin follows a circular projection [35]. The Red-tailed Hawk and American Kestrel both have 33° of binocular overlap at the 90° elevation at rest (Fig. 1d, f).

The maximum width of the binocular field at rest occurred at elevation 90° in both Red-tailed Hawks and American Kestrels (Fig. 2). However, in the Cooper's Hawk, the maximum binocular width was at elevation 70°, which made the binocular field noticeably wider above than below the plane of the bill (Fig. 1b, Fig. 2). Across all recorded elevations, the overall width of the binocular field differed significantly among species (Table 1), with Cooper's Hawks having overall wider binocular fields (28.60±1.78°) than Red-tailed Hawks (21.83±2.16°; t _12_ = 2.42, P = 0.032) and American Kestrels (22.29±1.13°; t _12_ = 2.99, P = 0.011). We also found a significant difference in the width of the binocular field among species depending on elevation (Table 1, Fig. 2). Furthermore, the vertical extent of the binocular field in the median sagittal plane varied significantly among species (F_2,11_ = 17.18, P<0.001; Figs. 2), being larger in the American Kestrel (112.50±2.65°; t_11_ = 5.75, P<0.001) and the Cooper's Hawk (110.00±4.32°; t_11_ = 4.36, P = 0.001) than in the Red-tailed Hawk (83.33±4.32°).

**Table 1:** Differences in the average width of the binocular field, blind area, and pecten among Red-tailed Hawks, Cooper's Hawks, and American Kestrels, considering the effects of elevation around the head.

	F	d.f.	P
**Binocular field**			
Species	28.71	2, 12	<0.001
Elevation	41.75	13, 115	<0.001
Species×Elevation	2.35	20, 115	0.003
**Blind area**			
Species	106.07	2, 12	<0.001
Elevation	49.77	17, 122	<0.001
Species×Elevation	1.34	25, 122	0.151
**Pecten**			
Species	0.04	2, 4	0.962
Elevation	4.32	8, 39	<0.001
Species×Elevation	3.25	10, 11	0.033

Results from General Linear Models.

Red-tailed Hawks and Cooper's Hawks have blind areas starting around the 30° elevation, while that of American Kestrels does not start until past the 0° elevation (Fig. 2). At the 90° elevation, Red-tailed Hawks have the widest blind area, Cooper's Hawks, the narrowest, and American Kestrels, intermediate values (Fig. 1 d–f). Across all recorded elevations, the overall width of the blind area at rest estimated by the model varied significantly among the three species (Table 1), with Red-tailed Hawks having wider blind areas (54.66±2.70°) than American Kestrels (35.11±1.88°; t_12_ = 5.93, P<0.001) and Cooper's Hawks (33.94±2.89°; t_12_ = 5.24, P<0.001). We also found a significant elevation effect (Table 1). In most elevations from behind the head (270°) to almost the top of the head (350°), Cooper's Hawks had a narrower blind area than American Kestrels; however, in the area above the head (from 350° to approximately 20°) American Kestrels had a narrower blind area than Cooper's Hawks (Fig. 2).

Finally, across all elevations, the width of the pecten did not vary significantly between species (Table 1): Red-tailed Hawk (20.50±1.36°), Cooper's Hawk (21.88±1.91°), and American Kestrel (23.47±0.80°). However, we found significant differences in the width of the pecten among species depending on the elevation (Table 1; Fig. 1a–c).

### Degree of eye movement and visual fields

Red-tailed Hawks and Cooper's Hawks exhibited considerably larger degree of eye movement in relation to American Kestrels (Fig. 3). The overall maximum degree of eye movement was recorded at elevation 270° in Cooper's Hawks, and 290° in Red-tailed Hawks and American Kestrels (Fig. 3). Eye movements varied significantly among species considering all elevations (F _2,13_ = 167.56, P<0.001), with all pair-wise comparisons being significant (t _13_ varied from 6.77 to 16.99, P<0.001). Cooper's Hawks had the largest degree of eye movements (8.35±0.32°), Red-tailed Hawks had intermediate values (4.90±0.40°), whereas American Kestrels had the lowest values (0.88±0.30°). Eye movement varied across elevations (F _23, 215_ = 6.93, P<0.001), but we also found that this variation across elevations differed among species (F _44, 215_ = 2.34, P<0.001, Fig. 3). For both Red-tailed Hawks and Cooper's Hawks, eye movements were greater around the horizontal plane in front and at the back of the bird's head (Fig. 3). However, Cooper's Hawks had a greater degree of eye movement in front of the head than Red-tailed Hawks (Fig. 3).

**Figure 4:**
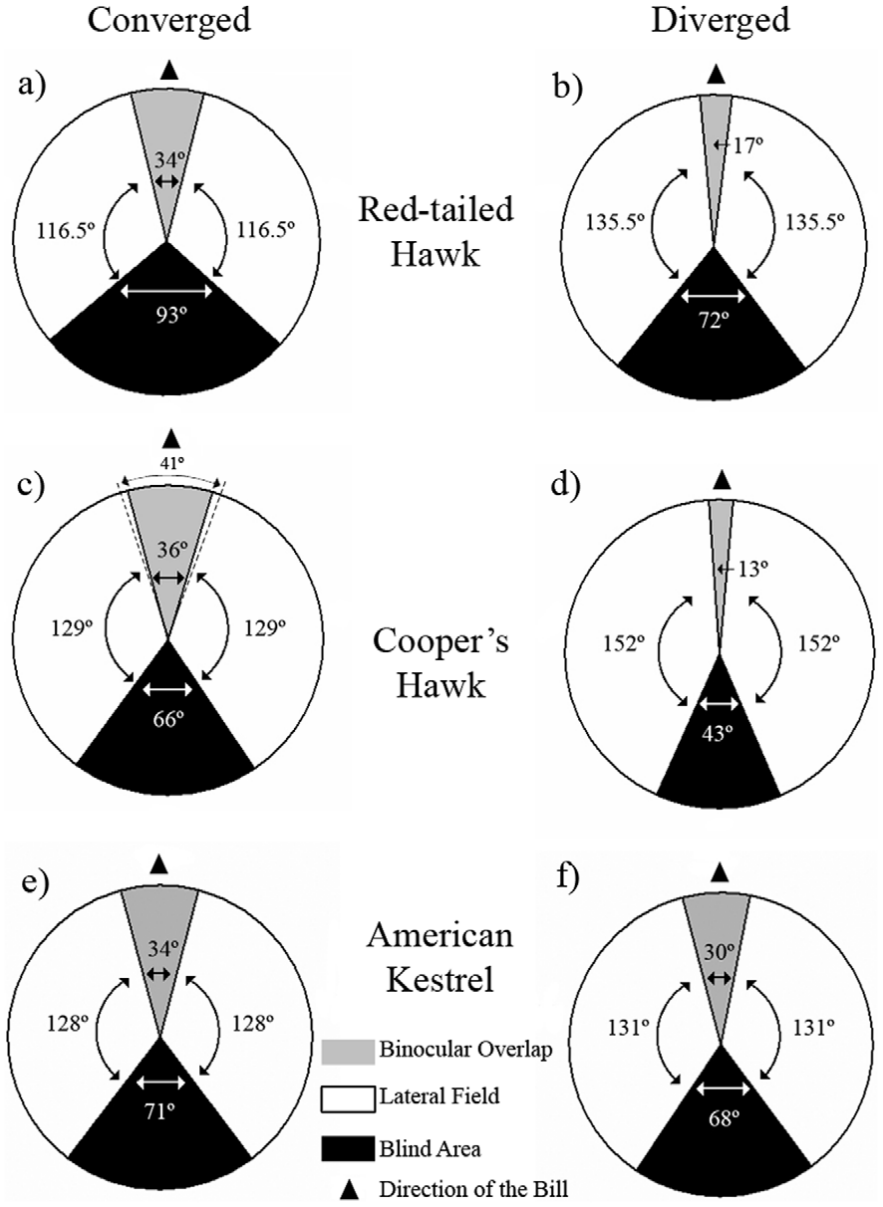
Visual fields of three diurnal raptors with the eyes converged and diverged. Horizontal sections through the horizontal plane (90°–270°) showing the visual field configuration of (a, b) Red-tailed Hawks, (c, d) Cooper's Hawks, and (e, f) American Kestrels. Charts represent the average retinal fields when the eyes were fully converged (eyes rotated fully forward; a, c, e), which maximizes the size of the binocular and blind areas, and fully diverged (eye rotated fully backward; b, d, f), which minimizes the size of the binocular and blind areas. The dotted lines in the Cooper's Hawk (c) chart represent the extrapolated binocular field assuming that the retinal margin follows a circular projection (see text).

Eye movements modified the configuration of the visual fields considerably in both hawk species at the horizontal plane. For Red-tailed Hawks, converging the eyes increased the binocular field and blind area by 3% and 13% (Fig. 4a), respectively, in relation to the eyes-at-rest position (Fig. 1d); whereas diverging the eyes, decreased the binocular field and blind area by 49% and 12% (Fig. 4b), respectively, in relation to the eyes-at-rest position (Fig. 1d). When Cooper's Hawks converged their eyes, the bill got in the way of our measurements, suggesting they can also see the tip of their bills in this eye position. We then estimated the size of the Cooper's Hawk binocular field with converged eyes at elevation 90° as 41° (Fig. 4c). This estimated value was extrapolated from the elevations right above and below 90° where the bill did not obstruct our measurements, assuming that the retinal margin follows a circular projection [35]. Based on these estimates of the binocular field of Cooper's Hawks, converging their eyes increased the size of the binocular field and blind area by 5% and 10% (Fig. 4c), respectively, in relation to the eyes-at-rest position (Fig. 1e); whereas diverging their eyes, decreased the binocular field and blind area by 64% and 28% (Fig. 4d), respectively, in relation to the eyes-at-rest position (Fig. 1e). Finally, for American Kestrels, converging their eyes increased the size of the binocular field and blind area by 3% and 4% (Fig. 4e), respectively, in relation to the eyes-at-rest position (Fig. 1f); whereas diverging their eyes, decreased the binocular field by 9% without affecting the blind area (Fig. 4f), in relation to the eyes-at-rest position (Fig. 1f).

### Orbit orientation

We did not find significant differences in orbit convergence among species (F _2,6_ = 3.71, P = 0.089). Additionally, the rank order of the non-significant variation in orbit convergence was not associated with the rank order of the variation in binocular visual field overlap. American Kestrels showed the highest (26.17°±1.47°) degree of orbital convergence, meaning that their eyes faced more forward towards the bill. Red-tailed Hawks had the lowest orbit convergence (21.03°±1.25), meaning their eyes faced more toward the sides of the head. The orbital convergence of Cooper's Hawks was intermediate (23.07°±1.29°).

## Discussion

Our study found between-species differences in visual fields and degree of eye movement in diurnal raptors that suggest some sensory specializations to gather visual information. We discuss these specializations in the context of the ecology of each species and then draw some comparisons.

Of the three species, the Red-tailed hawk has the narrowest binocular area at the horizontal plane and the widest blind area. This configuration suggests that the lateral, rather than the binocular, visual fields may be key in visual information gathering about prey [36]. Given that this species scans from high vantage points (>10 m [37]), we suggest that prey detection takes place at far distances [38] using the lateral visual fields with high acuity because they encompass the central fovea [39]. Visual acuity can be considered the highest in the Red-tailed hawks given its eye size [40]. Red-tailed Hawks (eye axial length, 22.80 mm) have the largest eyes compared to Cooper's Hawks (eye axial length, 18.00 mm) and American Kestrels (eye axial length, 11.95 mm) [41]. Head movement patterns in Red-tailed Hawks actually underscore the relevance of the lateral visual fields while perching as individuals turn their heads slowly and fixate on a visual target sideways rather than straight [39], [42]. Additionally, visual coverage appears limited in Red-tailed Hawks due to their wide blind areas. Large blind areas could be particularly relevant when the animals perch in open areas to reduce glare effects from the sun, which are more prevalent in species with relatively larger eyes [43].

The Cooper's Hawk has the widest binocular field of the three species, with a narrow blind area behind its head. Cooper's Hawks inhabit visually complex and closed habitats, which may require more binocular overlap to enhance prey detection through the vegetation [44], [45]. Changizi and Shimojo [44] predicted that in vegetation-cluttered habitats, species with interpupillary distance larger than the average leaf size would show wide binocular fields. The rationale is that the two monocular views would provide images different enough to allow the animal in front of a layer of leaves to *look around* the leaves in the *foreground* and actually increase visual coverage of the *background*. Although there is some evidence in mammals supporting this hypothesis [44], Martin [36] recently suggested that avian binocular vision may be more involved in the physical capture of prey at close quarters. Thus, the avian binocular field is proposed to control for the position of the bill and the timing of its opening while approaching a target [36]. The fact that Cooper's Hawks can inspect visually the tip of their bills to probably enhance prey handling supports the hypothesis on the control of bill position [see another example in 11]. The wider binocular field of Cooper's Hawks above the plane of the bill may also provide more spatial information on approaching prey targets. This may be important when Cooper's Hawks fly very low using vegetation or artificial elements (e.g., buildings) as cover before surprising their prey [46], which are often above the bird's head. Overall, the Cooper's Hawk's quick sideways head movements [42], along with its reduced blind area and large degree of eye movements, suggests that this species may benefit by quickly shifting its visual fields in closed habitats to increase visual coverage and detect prey.

American Kestrels have binocular fields of intermediate size. Our estimates of the width of the binocular field were similar to those by Frost et al. [47], who used a different technique. Fox et al. [48] suggested that this species uses stereopsis for binocular depth perception, particularly at close distances from visual targets [47], which may help locate small and cryptic invertebrate prey items while hovering [49]. However, perching American Kestrels fixate on more distant objects with their lateral areas [47], also moving their heads slightly upwards or sideways while keeping their bodies stationary and their bills facing in the same direction [42]. These perching head movement patterns may increase the detection of prey by providing depth cues through motion parallax [50]. Although the overall size of the American Kestrel blind area is intermediate in relation to the other two species, it has the narrowest blind area right above its head, which may be related to predator surveillance [51]. The American Kestrel is relatively small and has several predators, including Red-tailed Hawks, Cooper's Hawks, Northern Goshawks (*Accipiter gentilis*), Peregrine Falcons (*Falco peregrinus*), and Barn Owls (*Tyto alba*) [18]. Interestingly, American Kestrels have been reported to have spots at the back of their heads resembling eyes, which could be used to confuse a predator [52].

American Kestrels showed a very low degree of eye movement, which runs counter the results obtained by Frost et al. [47] and Pettigrew [53]; however, their measurements were done on anesthetized animals. This raises interesting questions about the physiological control of the extraocular muscles in this species, and even in birds in general. Preliminary evidence shows a lack of myosin heavy chain isoforms in the extraocular muscles of American Kestrels, but up to six distinct isoforms in Red-tailed Hawks and Cooper's Hawks (C.T. O'Rourke, B.C. Rourke, E. Fernández-Juricic, unpublished data). This degree of variability might influence the contractile properties of the eye muscles in different species, although further research is warranted. Phylogeny could play a role in the between-species differences in eye movements, as Red-tailed Hawks and Cooper's Hawks (Accipitridae) are both more closely related to each other than to American Kestrels (Falconidae). However, Short-toed Eagles also belong to the Accipitridae Family and do not show noticeable eye movements [20].

Between-species differences in the degree of eye movement could also be related to prey hunting strategies. Eye movements can prevent retinal blur [54] by tracking a moving target and compensating for the difficulty of moving the head during an attack flight [55]. Both Red-tailed Hawks and Cooper's Hawks primarily hunt small- to medium-sized birds and mammals that can engage in fast evasive action [56], which may require rapid eye movement adjustments. Eye movements have been found in other predatory avian species, such as Great Cormorants and Herons, which also hunt by pursuing evasive aquatic prey [12], [29]. However, American Kestrels feed primarily on invertebrates (e.g. beetles and crickets [18], [56]), and instead of engaging in pursuit attacks, they make quick stooping attacks from perches or by hovering above prey item, drop down, then pin it against the ground, which may require that the image is as stable as possible. A similar attack strategy is also used by the other diurnal raptors with negligible eye movements (Short-toed Eagles) when hunting snakes [20], [57], which may not be evasive enough to require a large degree of eye movement.

A recent comparative study suggested that the degree of orbital convergence in birds is associated with the degree of binocular overlap [27]. The rank order of orbital convergence (American Kestrel > Cooper's Hawk > Red-tailed Hawk) did not exactly match the rank order of the binocular overlap at rest (Cooper's Hawk > American Kestrel > Red-tailed Hawk), even with converged or diverged eyes. However, our three studied species had degrees of orbital convergence within about 5° of each other, which may suggest that orbit orientation may be too coarse a measure to detect subtle differences within these Falconiformes. Orbital convergence is a good indicator of binocular overlap in mammals [58], but further studies including avian species with different degrees of eye movement are necessary to establish the relationship between skull morphology and visual field configuration.

Raptor vision has intrigued zoologists for a long time [59], particularly considering the extensive research done in owls [60]–[62]. The raptorial visual system has been usually generalized as having large eyes, high acuity, two visual foveae, relatively large binocular areas (but see [20]), small blind areas, and relatively little degree of eye movement [47], [59]. Our findings suggest a larger degree of variability in visual field configuration and eye movement within birds of prey. Although previous studies have shown low degrees of eye movement in raptors (1.5°, Great Horned Owl *Bubo virginianus* [63]; 2.8°, Little Eagle *Haliaetus morphnoides* [64]) our study shows that some species of diurnal raptors are capable of a larger degree of eye movement, which can change the size of the lateral and blind areas by converging or diverging the eyes. Therefore, eye movement in hawks gives flexibility in visual field configuration. Additionally, we found that the blind areas of some raptors can be similar in size to those of some prey species (Brown-headed Cowbird *Molothrus ater*, Mourning Dove *Zenaida macroura* [28]) but are still generally large in comparison with many other birds studied to date [21].

We conclude that (1) diurnal raptors show inter-specific variability in visual traits involved in gathering information about prey; (2) differences in visual field configuration appear associated with prey searching strategies and visual obstruction in open and closed habitats; (3) between species variations in eye movement appear related to foraging strategies; and (4) the size of the binocular and blind areas in hawks can vary substantially due to eye movements. The degree of inter-specific variability in visual field configuration and degree of eye movement is consistent with behavioral variations in scanning strategies, such as the patterns of head movement while perching (see [42]). This information can be incorporated into predator-prey interaction models [31] to better establish the probabilities with which predatory species with different visual strategies would detect and visually track prey and to study the evolution of some anti-predator strategies.
